# Treatment of Burns

**Published:** 1888-09

**Authors:** 


### TREATMENT OF BURNS.

Nikolsky claims to have had excellent success in the treatment of burns of the second degree with fomentations of the following:

Tannin.
Alcohol (95) aa 4 grammes.
Rectified sulphuric ether, 30 grammes.

The dressings are repeated two or three times a day. After the ether evaporates there remains a fine pellicle of tannin that causes prompt sedation of the pain and inflammation. The cure takes place much more rapidly than under the employment of collodion and other remedies in common use. The first dressing should be preceded by an antiseptic washing of the affected parts. The bullæ are punctured to allow the serum to escape, and the denuded surfaces are then powdered over with iodoform in a thin layer, and then dressed with the ether-tannin.—*Journal de Médicine de Paris*, May 20, 1888.

